# Mandibular prognathism caused by acromegaly – a surgical orthodontic case

**DOI:** 10.1186/1746-160X-5-16

**Published:** 2009-08-06

**Authors:** Martin Gosau, Corinna Vogel, Antonios Moralis, Peter Proff, Johannes Kleinheinz, Oliver Driemel

**Affiliations:** 1 Department of Cranio-Maxillo-Facial Surgery, University of Regensburg, Regensburg, Germany; 2 Institute of Pathology, University of Regensburg, Regensburg, Germany; 3 Department of Orthodontics, University of Regensburg, Regensburg, Germany; 4 Department of Cranio-Maxillofacial Surgery, University of Münster, Münster, Germany

## Abstract

A 22-year-old man presented for orthodontic surgery because of mandibular prognathism. Clinical symptoms suggested acromegaly, and diagnosis was verified by an endocrinologist as well as by radiograph. Bilateral mandibular prognathism often represents the first and most striking physical characteristic of acromegaly; usually, it is also the main reason why patients seek help from orthodontists or maxillo-facial surgeons. This case report recapitulates the clinical and histopathological findings in pituitary growth hormone (GH) adenomas and emphasises their importance in surgical orthodontic planning. Mandibular prognatism, macroglossia and abnormal growth of hands and feet represent strong indicators for the diagnosis of acromegaly. This disease and its complications not only affect the entire body but increase mortality if the pituitary gland tumour remains untreated.

## Introduction

Pituitary adenomas have a variety of clinical manifestations that are related to excessive hormone secretion by the tumour, hormone deficits by normal pituitary gland tissue and the expansion of tumour mass. Most patients have a 5- to 10-year history of changes in facial features before acromegaly is diagnosed [1,2]. Our case report recapitulates the clinical and histopathological findings in pituitary GH adenomas and associated acromegaly and emphasises their importance in surgical orthodontic planning.

## Clinical history

**Figure 1:**
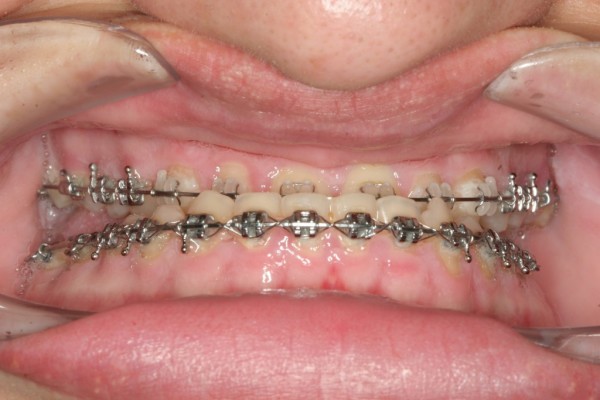
**Initial intraoral photograph showing Angle class III malocclusion**.

**Figure 2:**
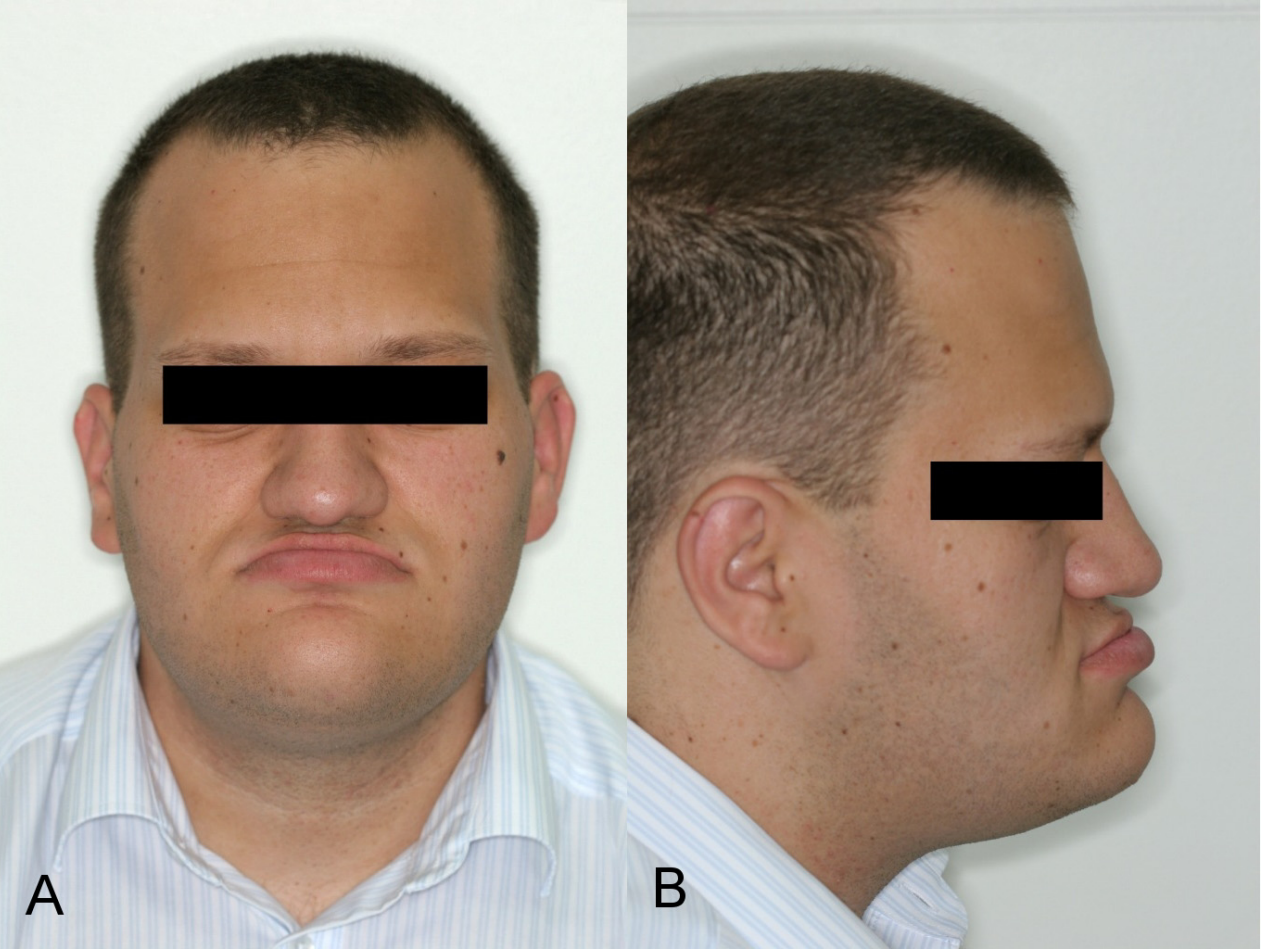
**Initial facial photographs (2a: frontal and 2b: lateral view)**.

**Figure 3:**
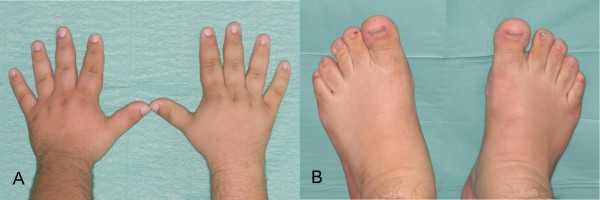
**Initial hands and feet photograph**.

A 22-year-old man was referred to our hospital for orthognathic surgery after 12 months of presurgical orthodontic treatment (fig. 1). Physical examination showed pronounced mandibular prognathism, a widened and thickened nose, prominent supraorbital ridges, thick and coarsened lips and marked facial lines (fig. 2). Intraorally, macroglossia was evident, impairing speech. The patient reported that mandibular prognathism had began to develop 4 years previously. Additionally, his shoe size and hand size had increased considerably during the last few months (fig. 3).

**Figure 4:**
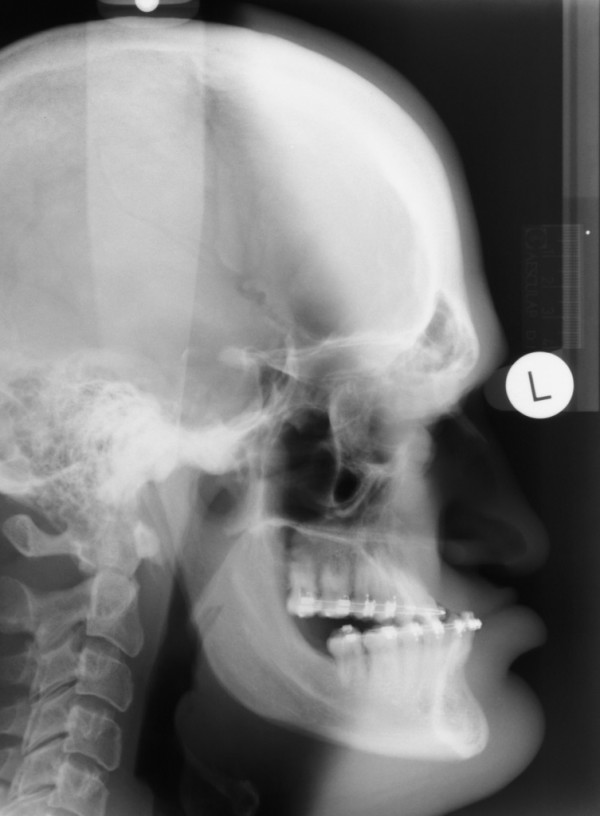
**Lateral cephalometric radiograph**.

**Figure 5:**
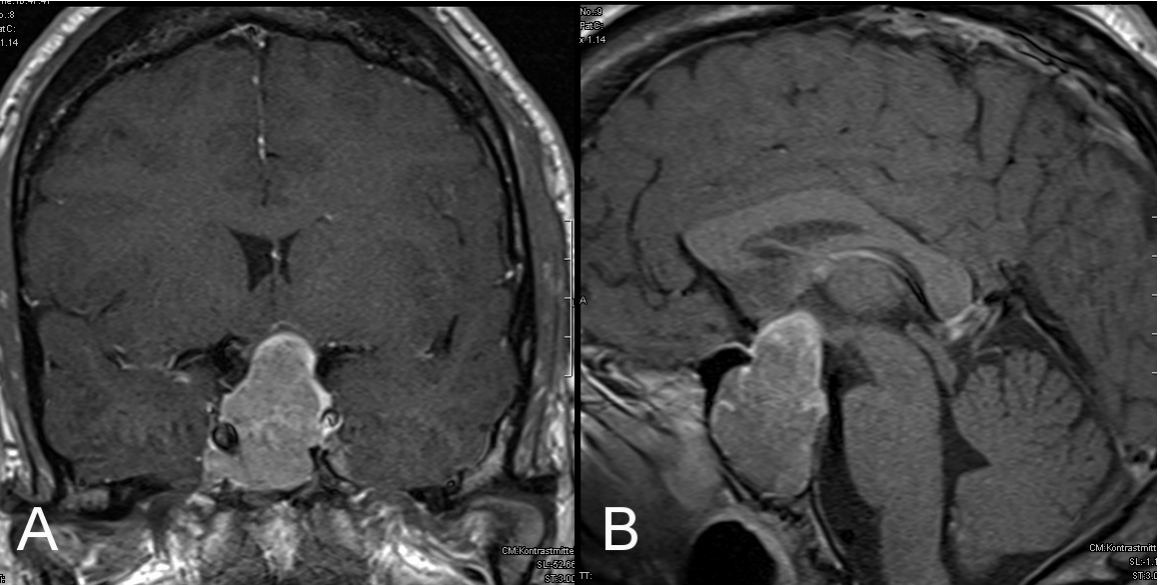
**MRIs of tumour site**. 5a. coronal 5b. sagittal: MRI scans show extensive growth in and above the sella turcica with a total volume of 4.7 × 2.9 × 2.2 cm^3^.

Lateral cephalometric radiograph examination showed massive mandibular prognathism, prominent supraorbital ridges and an enlarged sella turcica (fig. 4). Additionally, magnetic resonance imaging (MRI) scans confirmed the expansively growing tumour mass within and above the sella turcica (fig. 5). Furthermore, the entire calvarian bone was thickened, thus confirming the provisional diagnosis of acromegaly. The patient had been referred for examination by an endocrinologist. Diagnosis of a pituitary GH producing macroadenoma was confirmed as well as reactive hyperprolactinaemia and deficiency of the gonadotropin axis. Diabetes mellitus was not found.

Endocrinological examination showed increased levels of the insulin-like growth factor-I (IGF-I) (627.0 ng/ml; norm: 117.0 to 329.2 ng/ml), increased prolactin (63.69 μg/l; norm: 2.10 to 17.7 μg/l) and depressed testosterone levels (0.84 μg/l; norm: 2.41 to 8.30 μg/l). Ultrasonography showed hepatosplenomegaly and an enlarged left kidney. In addition, colonoscopy showed dilatation of the colonic lumen. Ophthalomological screening showed a large bi-temporal visual field defect due to tumour compression of the optic chiasm. The patient underwent trans-sphenoidal surgery with complete tumour resection.

**Figure 6:**
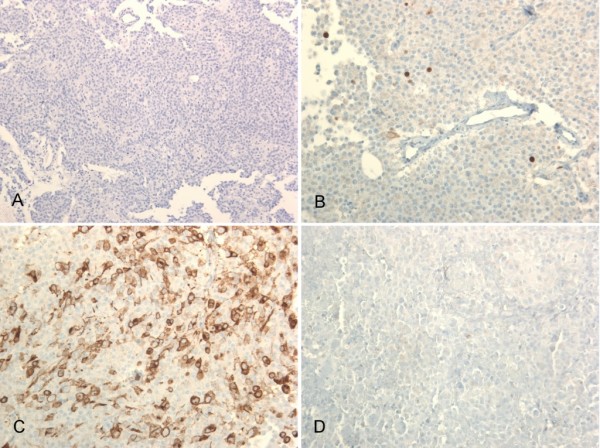
**Highly vascularised pituitary adenoma with diffuse (solid) growth pattern**. Higher magnification shows uniform cells with broad eosinophilic cytoplasm and round to oval nuclei (Hematoxylin and Eosin, A 100×). The proliferation index is very low with approximately 3% of cells showing immunoreactivity against MiB-1 (B 200×). Some tumour cells show immunopositivity for prolactin in the peripheral areas of the cytoplasm (C 200×). However, no immunoreactivity was present that provided an antibody against the human growth hormone (hGH, D 200×).

Histological examination showed a highly vascularised pituitary adenoma with a diffuse (solid) growth pattern. Higher magnification showed uniform cells with broad eosinophilic cytoplasm and round to oval nuclei (fig. 6a). The proliferation index was very low with approximately 3% of cells showing immunoreactivity against MiB-1 (fig. 6b). Parts of the tumour cells showed immunopositivity for prolactin in peripheral parts of cytoplasm (fig. 6c). However, no immunoreactivity was present that provided an antibody against the human growth hormone (fig. 6d).

Reestablishment of endocrine balance would be followed by mandibular osteotomies to reestablish facial harmony and Angle class I occlusion.

## Discussion

Since symmetrical mandibular prognathism can be part of syndromal growth abnormality, this disease is usually noticeable shortly after birth or in early childhood. Non-syndromal growth abnormalities of the jaws are mainly due to genetic or unknown factors. The third group are acquired growth abnormalities of the jaws. When symmetrical bilateral mandibular prognathism is noticed, the diagnosis of acromegaly should always be taken into consideration [3].

Acromegaly is a rare disease with a prevalence of 40 to 70 cases per million people in the population and an annual incidence of 3 to 4 new patients per million [4,5]. Owing to its insidious onset, acromegaly is often diagnosed late (4 to over 10 years after onset) at an average age of about 40 years. The disease affects men and women equally [6,7].

In our patient, the diagnosis of acromegaly was considered because of the patient's manifestations and symptoms at first presentation. Mandibular prognathism is amongst the most commonly found oral manifestations of acromegaly and it was the main reason for the patient to seek orthodontic treatment. Enlargement of the lips and the tongue, such as in our patient, may impair swallowing, chewing and speaking. Frontal bossing, prognathism, macroglossia and an increased size of hands and feet have often been described in the literature as cosmetic changes associated with GH adenomas [2,8-10]. All these changes were present in our patient. Lateral cephalometric radiograph examination of our patient showed massive mandibular prognathism, prominent supraorbital ridges and an enlarged sella turcica. Patients with acromegaly usually exhibit enlargement of all parts of the neurocranium and orofacial bones except the maxilla. The mandible usually shows the biggest enlargement, and the ramus is more affected than the body of the mandible [11,12]. The enlargement of the sella turcica caused by the tumour expansion of the pituitary gland is a striking manifestation that is detectable on lateral cephalometric radiographs in almost every patient with acromegaly [5,11,13]. Other symptoms reported by our patient included fatigue, daytime somnolence and joint pain.

Decreased energy, osteoarthritis and somnolence occur in about 50% of patients [2,9]. Patients also exhibit cardiovascular hypertension, congestive heart failure and impaired glucose metabolism, but these diseases were not present in our patient. Diabetes mellitus, sleep apnea (90%), lumbar stenosis and carpal tunnel syndrome (20%) are other possible manifestations of GH adenomas [1,7,14,15]. In adolescents, GH excess manifests clinically as acromegaly and gigantism if the onset of the disease occurs before the closure of the epiphyseal plates [8,10,16].

Adenomas that grow upwards or expand massively, such as in our patient, may compress the optic chiasm. Such a compression may lead to visual field defects, which begin in the superior temporal sectors and then progress to bitemporal hemianopsia. Persistent compression may lead to blindness. Routine assessment of visual fields and acuity is therefore essential.

Measurement of GH response to glucose load is the standard diagnostic test. An increase in the serum concentration of IGF-I, the main GH dependent growth factor, confirms this diagnosis [2,10]. MRI has emerged as the imaging modality of choice for evaluating pituitary glands [8]. Some adenomas are mixed; mixed GH- and prolactin (PRL)-secreting adenomas occur frequently (25%). Therefore, elevated prolactin levels, such as in our patient, are not unusual.

Available treatment options include surgery, medication and radiotherapy [16]. Trans-sphenoidal surgery is regarded as the first-line treatment of acromegaly [9]. Depending on the tumour size, long-term remission is achieved in 50% [9] to 67% [10,17] of patients, but safe removal of all tumour tissue can be difficult. Microadenomas (less than 10 mm in diameter) are more amenable to cure. Preoperative GH concentration seems to be the best indicator for successful treatment and a permanent reduction of GH and IGF-I [18]. If surgery fails to cure acromegaly, medical treatment with somatostatin analogues is recommended rather than radiotherapy [10]. Regardless of the initial choice of treatment, patients should be followed up indefinitely [2,10,16]. Our patient was treated with trans-sphenoidal surgery, which adequately reduced GH and IGF-I levels up to six months postoperatively.

After a permanent reduction of GH and IGF-I levels, the enlarged soft tissue regresses gradually [19]. However, only little improvement in bone changes may be expected. After a stable reduction of GH and IGF-I levels of at least two years, bimaxillary osteotomy may be conducted for orthognatic correction and reduction of supraorbital ridges in one single procedure. However, late relapses may occur due to a recurrence of pituitary gland adenoma [12,20,21].

## Conclusion

Acromegaly is a rare disease that is responsible for bilateral mandibular prognathism in adults. Excess of growth hormone and local tumour growth of the pituitary gland affect the entire body and increase mortality. In most patients, this disease is diagnosed far too late. Orthodontic and maxillo-facial surgeons dealing with orthognatic surgery should be well aware of this disease as patients usually present with them first because of striking mandibular growth. In case of mandibular prognatism in combination with enlargement of lips, nose, tongue, hands and feet followed by visual field defects, sleep apnea, decreased energy and osteoarthritis, every clinician should consider the diagnosis of acromegaly. Although acromegaly is a rare disease, its symptoms are striking and can hardly be misconstrued.

## Competing interests

The authors declare that they have no competing interests.

## Authors' contributions

GM and MA analysed the patient's history, reviewed all patient data and drafted the manuscript. VC carried out the histological analysis, wrote the histological part of the paper and contributed to the writing of the final version. DO, PP and KJ were involved in revising the article. Each author reviewed the paper for content and contributed to the writing of the manuscript. All authors approved the final report.
